# Hybrid Analytical and Simulation-Based Approach for Workspace Verification of a Pneumatic Upper Limb Exoskeleton

**DOI:** 10.3390/s26113308

**Published:** 2026-05-22

**Authors:** Nikita Mayorov, Daniil Teselkin, Denis Dedov, Artem Obukhov

**Affiliations:** Laboratory of VR Simulators, Tambov State Technical University, Tambov 392000, Russiadteselk@mail.ru (D.T.); hammer68@mail.ru (D.D.)

**Keywords:** robotic systems, upper limb exoskeleton, manipulator workspace, pneumatic drive, product of exponentials method, collision detection, virtual reality

## Abstract

The design of active pneumatic upper limb exoskeletons is complicated by the challenge of reliably determining a kinematically safe workspace. Existing analytical kinematic methods are not sufficient to predict geometric collisions between elements of closed kinematic chains, which poses risks of mechanical damage and threats to user safety during exoskeleton operation. This paper proposes a hybrid algorithm for verifying the workspace of a pneumatic exoskeleton, combining analytical modelling in MATLAB R2020b based on the Product of Exponentials (PoE) method with high-performance static simulation in the Unity environment. At the initial stage, a discrete set comprising 758 million positions of the upper exoskeleton manipulator was generated. Subsequently, a multithreaded two-stage filtering process was implemented: analytical verification of rod stroke limits and angular constraints, followed by the detection of physical intersections of solid-state meshes using the PhysX engine. The results indicate that while the analytical model filters out 99.6% of invalid configurations. Yet, among the remaining positions—formally correct from a mathematical standpoint—up to 50% lead to critical geometric collisions or breaks in the kinematic chain. The computational efficiency of the proposed architecture enabled full static workspace verification in under 20 min. A reachable zone topology was established, revealing pronounced asymmetry and the presence of a “manoeuvrability core” in the user’s anterior hemisphere. The developed algorithm generates a verified set of kinematically safe exoskeleton states, providing a foundation for the kinematic safety layer of a hierarchical control system. These findings demonstrate the necessity of complementing analytical kinematics with physical collision detection when designing hybrid kinematic mechanisms, and the approach can be applied to verify collision-free movement trajectories in various robotic systems. The approach can be applied to verify collision-free movement trajectories in simulation, with physical validation deferred to future work.

## 1. Introduction

The integration of robotic systems into the clinical practice of medical rehabilitation and complex professional training environments represents a rapidly evolving frontier in modern engineering. Such systems, employing active upper limb exoskeletons, significantly accelerate the recovery of lost motor functions in patients following acute cerebrovascular accidents. Concurrently, exoskeleton systems facilitate the emulation of mechanical loads and interactions with virtual objects within specialised professional training frameworks.

The development of hardware-software complexes in this class is intrinsically linked to the requirement of ensuring unconditional safety within the physical interaction of the ‘human–machine’ loop (ergatic system). Traditionally, the mathematical apparatus of forward and inverse kinematics serves as the foundational framework for the synthesis of safe kinematic trajectories and control algorithms. Analytical methods allow for the high-precision translation of workspace target coordinates into the generalised coordinates of the manipulator joints. However, engineering practice demonstrates that reliance solely on kinematic models proves insufficient when designing complex mechanisms featuring spatial parallel kinematics. Exoskeletons driven by an assembly of pneumatic cylinders epitomise this class of systems.

A fundamental limitation of the analytical approach lies in the fact that the model operates with abstract geometric vectors and axes of rotation, completely disregarding the physical volume of solid-state structural elements. During the operation of an exoskeleton characterised by closed kinematic chains (four-bar linkage mechanisms, intersecting planes of rod motion), there is a high risk of internal geometric collisions—unintended physical impacts between the structural frame links and actuator housings. Consequently, a solution to the inverse kinematics problem that is formally correct from a mathematical standpoint may prove physically unrealisable in practice. This inevitably leads to mechanism jamming, damage to pneumatic drives, or the creation of hazardous situations for the user.

Thus, a comprehensive verification of system functionality and the determination of the true boundaries of the safe workspace require full-scale simulation modelling, ensuring collision detection across the entire manifold of reachable configurations. Utilising classical dynamic simulation environments (such as Gazebo, Webots, or Simscape Multibody) for these purposes entails unacceptably high computational costs. The architecture of the solvers employed therein—namely Linear Complementarity Problem (LCP) solvers or Differential-Algebraic Equation (DAE) solvers—mandates the calculation of time integrators and mass matrices even for static spatial queries. This circumstance renders the rapid processing of hundreds of millions of discrete configurations, necessary for constructing an exhaustive operational safety matrix, virtually unfeasible.

To overcome this identified limitation, this paper proposes a systemic methodological approach that combines the analytical rigour of the Lie group mathematical apparatus with the high computational performance of modern 3D graphics engines, optimised for the multithreaded calculation of spatial geometry.

The scientific novelty and primary contributions of this study are as follows:

- A hybrid two-stage algorithm for the verification of kinematic states has been developed and formalised, combining preliminary analytical filtering based on the Product of Exponentials (PoE) method with subsequent high-performance simulation of geometric intersections, bypassing resource-intensive physical dynamics calculations.
- The mathematical apparatus of the PoE method has been adapted for the rigorous description of the forward and inverse kinematics of a specific linkage-pneumatic upper exoskeleton architecture, explicitly accounting for parametric constraints on maximum cylinder rod extension lengths.
- The critical divergence between the theoretical (analytical) and actual (physical) manipulator workspaces has been empirically demonstrated and quantitatively assessed, providing a rigorous justification for the mandatory inclusion of a geometric simulation stage in the design cycle of such mechatronic systems.
- A discrete matrix of safe states (verified workspace) has been synthesised, forming the fundamental algorithmic basis for the practical implementation of the control system and the seamless integration of the exoskeleton complex with interactive virtual reality scenarios.

It should be stressed that the present work targets the geometric and kinematic aspect of safety. Dynamic phenomena (inertial loads, friction, actuator compliance), as well as patient-specific anatomical constraints, lie outside the current scope and are acknowledged as limitations to be addressed in future stages of the control-system development.

The structure of this paper is organised as follows. Section 2 presents an analytical review of existing exoskeleton hardware platforms, mathematical methods for kinematics modelling, and available simulation environments. Section 3 is dedicated to a detailed description of the proposed methodology and the construction of the mathematical model. Section 4 presents the results of computational experiments performed using the developed algorithm. Section 5 contains a discussion of the obtained data and its comparison with results from related studies, while Section 6 summarises the main conclusions of the work.

## 2. Related Work

### 2.1. Analysis of Upper Limb Exoskeletons

Active upper limb exoskeletons employ electric, pneumatic, or hydraulic actuators to assist movement during neurorehabilitation or immersive training, in contrast to passive systems that merely redistribute loads. Most modern platforms provide several degrees of freedom and support multiple control modes—passive, assistive, active, and resistive—to accommodate different therapeutic needs [1,2].

Among the available actuator technologies, pneumatic drives, such as those used in the Pneu-WREX system, are particularly well suited for rehabilitation applications [3,4,5]. Their high power density and inherent mechanical compliance enable the mechanism to act as a physical damper, safely absorbing sudden spastic contractions. In addition, pneumatic cylinders can maintain static loads against gravity for extended periods without the overheating problems common in electromechanical transmissions [6].

Regardless of the specific kinematic architecture, the control system of a rehabilitation exoskeleton must reliably handle three interrelated tasks:

1. Kinematics and trajectory tracking. Cartesian coordinates of the target point must be transformed into joint coordinates without entering singular configurations. Although some contemporary systems augment classical inverse kinematics with predictive or deep-learning components to compensate for muscle resistance and load variations [7], such “black-box” methods lack the transparency and determinism required for safety-critical applications. The problem is further complicated for pneumatic systems by the highly nonlinear compressibility of the working medium.
2. Hardware and software safety. A hierarchical safety loop is mandatory [8]. It should enforce limits on angular velocities and accelerations to protect the patient’s joints and ligaments, continuously monitor the pressure in pneumatic chambers, and trigger an emergency force release if abnormal resistance, e.g., spasticity, is detected. Equally important are geometric constraints that prevent the mechanism from exceeding the allowed range of motion. It should be clarified that muscle spasms constitute a force disturbance, not a kinematic command. If a spasm attempts to push the exoskeleton beyond the verified workspace, the force-constraint loop described above will detect the abnormal pressure rise and trigger an emergency pressure release, thereby preventing structural damage. Thus, the kinematic safety envelope works in concert with the force-monitoring layer to handle such events.
3. Interaction management and active compliance. When the exoskeleton is integrated with a virtual environment [9], the control architecture must support admittance control [10]—dynamically modulating the mechanical impedance so that the device yields to the patient’s voluntary efforts while providing haptic feedback. Electromyographic signals are often employed as an additional biological input [11,12] to further synchronise the patient’s intention with the assistive force.

The foregoing analysis underscores that the geometric constraint—ensuring that all components of the closed kinematic chain remain free of internal collisions—cannot be satisfied solely by real-time joint- or velocity-limiting algorithms. Instead, it demands an offline, exhaustive verification of the safe workspace, which serves as the foundation for the trajectory planner at the top level of the multi-loop control hierarchy.

### 2.2. Analysis of Mathematical Modelling Methods for Manipulator Kinematics

The classical Denavit–Hartenberg (DH) formalism [13] attaches local coordinate frames to each link and uses four parameters per joint. Its main drawback is the appearance of parametric singularities when consecutive joint axes become parallel or intersect. The Product of Exponentials (PoE) method, grounded in screw theory and Lie groups [14,15], avoids this problem: twists are invariant to the current configuration, eliminating the need to redefine link frames, and singularities are confined to coordinate representations rather than the underlying geometry. PoE also treats revolute and prismatic pairs uniformly and allows the Jacobian to be constructed directly from twist components without explicit differentiation. These properties have led to its widespread adoption in modern robotics [16,17].

The group of rigid body motions in three-dimensional Euclidean space ${\mathbb{R}}^{3}$ is represented by a mapping $g:{\mathbb{R}}^{3}\to {\mathbb{R}}^{3}$ having the form $g\left(x\right)=Rx+p$, where $R\in SO\left(3\right)$ is a rotation matrix (an element of the special orthogonal group) and $p\in {\mathbb{R}}^{3}$ is a displacement vector. An element of the special Euclidean group $g\in SE\left(3\right)$ can be written as a 4 × 4 matrix:

(1) $$g={\left[\begin{array}{cc} R & p\\ 0 & 1 \end{array}\right]}_{\left(4\times 4\right)},$$

The Lie algebra $\xi \in se\left(3\right)$ is the tangent vector to the identity element of the group $SE\left(3\right)$. The rotation of a revolute kinematic pair can be described as a generalised screw motion with zero pitch. The Lie algebra, which serves as the generator of such motion, has the following form:

(2) $$\hat{\xi }={\left[\begin{array}{cc} \hat{\omega } & v\\ 0 & 0 \end{array}\right]}_{\left(4\times 4\right)};\;\xi =\;{\left[\begin{array}{c} v\\ \omega \end{array}\right]}_{\left(6\times 1\right)},$$

(3) $$\hat{\omega }=\left[\begin{array}{ccc} 0 & -\omega_{3} & \omega_{2}\\ \omega_{3} & 0 & -\omega_{1}\\ -\omega_{2} & \omega_{1} & 0 \end{array}\right];\;\omega =\left[\begin{array}{c} \omega_{1}\\ \omega_{2}\\ \omega_{3} \end{array}\right],$$

(4) v=−ω×q,

where ω is the normalised rotation axis vector, and q is a point on the rotation axis.

Multiplying the Lie algebra by the rotation angle parameter and taking the exponent yields a one-parameter subgroup $SE\left(3\right)$, describing the rotation of a rigid body around the axis specified by this Lie algebra:

(5) $$g=e^{\hat{\xi }\theta },$$

where θ is the link rotation angle.

The forward kinematics of a serial spatial manipulator can be described using the product of exponentials:

(6) $$g\left(\theta \right)=e^{{\hat{\xi }}_{1}\theta_{1}}e^{{\hat{\xi }}_{2}\theta_{2}}\ldots e^{{\hat{\xi }}_{n}\theta_{n}}g_{0},$$

(7) $$e^{\hat{\xi }\theta }={\left[\begin{array}{cc} e^{\hat{\omega }\theta } & \left(I-e^{\hat{\omega }\theta }\right)\left(\omega \times v\right)+\omega \omega^{T}v\theta \\ 0 & 1 \end{array}\right]}_{\left(4\times 4\right)},$$

where n is the number of joints in the kinematic chain.

The matrix exponential of a skew-symmetric matrix in this case can be found using Rodrigues’ formula:

(8) $$e^{\hat{\omega }\theta }=I+\hat{\omega }\sin\theta +{\hat{\omega }}^{2}\left(1-\cos\theta \right).$$

For inverse kinematics, numerical optimization (e.g., the Newton-Raphson method [16]) does not guarantee deterministic computation time. However, to ensure control determinism and speed, analytical solutions are preferred if they can be obtained. In the PoE framework, an exact analytical solution can be obtained via the Paden-Kahan subproblems [15], bypassing iteration entirely and providing the fast, deterministic filtering required before geometric collision detection. However, even a perfectly formulated PoE model cannot, by itself, detect physical intersections between solid bodies; this necessitates the complementary simulation stage proposed in the present work.

### 2.3. Analysis of Simulation Environments for Exoskeleton Systems

Specialised simulators such as Gazebo, Webots, CoppeliaSim, and MATLAB Simscape are widely used for manipulator modelling. Yet, massively verifying static kinematic configurations for internal collisions exposes two fundamental architectural limitations, summarised in Table 1.

First, continuous-time coupling: Gazebo, Webots, and Simscape rely on LCP or DAE solvers that advance simulation time step by step. Collision queries cannot be executed independently of the dynamics clock without low-level kernel modifications or external service calls [18,19]; Simscape further requires penalty-based force integration. Consequently, these environments are inherently unsuitable for the rapid processing of hundreds of millions of discrete geometric states.

Second, insufficient parallel throughput: CoppeliaSim supports instantaneous intersection checks but executes them within a single thread, causing severe kernel blocking during mass verification. NVIDIA Isaac Sim offers GPU-accelerated batch detection, yet the overhead of PCIe transfers and continuous BVH reconstruction, together with the dependency on specialised hardware, negates the parallelisation advantage for the present task.

Among the evaluated platforms, Unity with the PhysX engine uniquely decouples the collision-detection subsystem from the dynamics integrator. By manually synchronising transforms and invoking Physics. ComputePenetration() inside multi-threaded C# Job System tasks, one can perform time-agnostic, high-throughput static intersection queries. This property directly addresses the core limitation of prior tools and makes Unity the ideal candidate for the geometric verification stage of the proposed hybrid analytical–simulation pipeline.

In summary, existing active upper-limb exoskeletons rely on analytical kinematics that cannot account for physical collisions in closed pneumatic chains, while mainstream simulation environments are architecturally unsuited for high-throughput static collision queries. No current approach combines a rigorous PoE-based analytical model with a scalable, time-agnostic geometric collision checker capable of processing hundreds of millions of configurations. The present work addresses this gap by proposing a hybrid pipeline that couples MATLAB-based PoE filtering with multithreaded PhysX collision detection in Unity, as described in the following section.

## 3. Materials and Methods

To resolve the identified challenges, this study proposes an approach that integrates two core components: an analytical mathematical model implemented in MATLAB (The MathWorks, Natick, MA, USA) and an interactive virtual environment developed in Unity (Unity Software Inc., San Francisco, CA, USA) (utilising C#) for verifying the movement trajectories of upper exoskeleton segments. The novelty of this approach lies in the formalisation of the kinematic model for the developed exoskeleton and the implementation of a sequential two-stage verification process, which eliminates the possibility of exoskeleton segments moving into physically inadmissible positions.

The research framework is structured as follows. Section 3.1 formalises the quantitative design requirements that the safe workspace must satisfy. Section 3.2 details the development of the upper limb exoskeleton’s kinematic model and defines its primary constraints. Section 3.3 describes the data processing algorithm for determining the permitted operational workspace, incorporating both kinematic constraint verification and simulation modelling within the virtual environment.

### 3.1. Quantitative Design Requirements

Before developing the mathematical model and the simulation pipeline, the quantitative requirements that the safe workspace must fulfil are formalised. These specifications are derived from the intended applications—post-stroke upper-limb rehabilitation and immersive professional training—and from the constraints of real-time control on embedded hardware.

1. Clinical and anatomical coverage. The verified workspace must encompass the functional range of motion of the human upper limb required for activities of daily living and post-stroke rehabilitation. Based on biomechanical standards, the target spatial zone is defined as a sphere of radius 1.00 m centred at the shoulder attachment point.
2. Computational resolution and determinism. To guarantee the absence of micro-collisions between discrete calculation steps, the spatial grid resolution must be strictly defined at 10 mm, and the angular orientation step must be no greater than 1°. This dense discretisation generates an initial configuration set exceeding 7.5 × 10^8^ states, ensuring that the discrete approximation faithfully represents the continuous workspace.
3. Algorithmic performance. Traditional dynamic simulators that compute forces and advance time steps require tens of hours to process discrete state matrices of this magnitude. The requirement for the proposed hybrid verification pipeline is to complete the exhaustive spatial analysis of the entire dataset (>7.5 × 10^8^ configurations) in under 1 h (<3600 s). This necessitates an architecture capable of processing at least 2 × 10^5^ geometric collision queries per second.

These quantitative metrics serve as the baseline for evaluating the computational efficiency and practical viability of the developed hybrid algorithm in the subsequent sections.

### 3.2. Upper Limb Exoskeleton Kinematics

This work does not aim at kinematic synthesis: the geometric parameters of the manipulator are predetermined by the existing mechanical design, which was developed on the basis of the Pneu-WREX rehabilitation robot [5]. The workspace evaluation requires solving the inverse kinematics problem—mapping the end-effector workspace coordinates to the joint angles and subsequently to the pneumatic cylinder lengths. At the same time, computing the required cylinder lengths from known angles involves a forward transformation that determines the positions of the cylinder attachment points on the links. The forward kinematic equations of the manipulator were derived using the Product of Exponentials (PoE) method.

Design parameters are given as nominal dimensions in millimetres; all angles are expressed in radians. Numerical values obtained from symbolic computations in MATLAB are rounded to four decimal places in the text for brevity, while the computational model employs full double-precision arithmetic.

Because the manipulator possesses a hybrid serial-parallel kinematic structure, not all joint angles are independent. To simplify the analysis, the known kinematic dependencies are directly substituted into the PoE formula, treating the chain as fully serial. Moreover, the pneumatic cylinders that act as actuators are not introduced into the PoE model as prismatic pairs. This considerably reduces the complexity of the system while preserving full kinematic equivalence with the physical mechanism.

Determining the admissible ranges of the angular coordinates $\theta_{i}$ is an important design step that ensures kinematic accuracy and interaction safety. The limits for the shoulder assembly angles—$\theta_{1}$ (horizontal shoulder offset) and $\theta_{3}$ (shoulder elevation/depression)—are established during the modelling stage based on the condition of avoiding mechanical contact between links. Constraining $\theta_{1}$ and $\theta_{3}$ effectively prevents collisions of the first link with the base and collisions among the links of the parallelogram mechanism. Because the collision-inducing limits of $\theta_{2}$ depend non-linearly on $\theta_{1}$, no explicit bounds are imposed on $\theta_{2}$ at this stage, which necessitates additional secondary intersection-verification algorithms.

A special role is played by the forearm rotation angle $\theta_{5}$, which is uniquely determined by the handle orientation angle $\theta_{5b}$. Unlike $\theta_{1}$ and $\theta_{3}$—rigidly defined by the manipulator geometry—the limits of $\theta_{5b}$ also depend on the anthropometric features of the upper limb. Due to the individual variability of biological parameters, precisely determining the boundaries of this range remains an open task. At the present stage of research, the limits for $\theta_{5b}$ are set on the basis of normal physiological ranges of motion with an added safety margin. However, these limits do not account for potential collisions involving the fourth pneumatic cylinder; admissible values of $\theta_{5b}$ depend non-linearly on $\theta_{3}$, which again necessitates an additional intersection check. No separate constraints are introduced for $\theta_{4}$ and $\theta_{5}$ because they are uniquely determined by $\theta_{3}$ and $\theta_{5b}$, respectively.

The angular coordinate constraints (in radians) are:

(9) $$\theta_{1}\in \left[-0.7505,0.7505\right];\theta_{3}\in \left[-0.6109,0.9250\right];\theta_{5b}\in \left[-0.3491,0.5236\right].$$

The admissible range of the linear coordinates (in mm), determined by the physical stroke lengths of the pneumatic cylinders and the geometry of their attachments to the links and frame, is:

(10) $$x_{p1}\in \left[352,502\right],\;x_{p2}\in \left[502,802\right],\;x_{p3}\in \left[277,352\right],\;x_{p4}\in \left[438,638\right].$$

For the parallelogram mechanism, the constraint $\theta_{4}=-\theta_{3}$ is incorporated by replacing the exponential of the corresponding joint axis for $\theta_{4}$ with $e^{\xi_{4}\left(-\theta_{3}\right)}$. For the four-bar linkage, the functional dependence $\theta_{5}\left(\theta_{5b}\right)$ is first derived, and the exponential of the last revolute joint is written as $e^{{\hat{\xi }}_{5}\theta_{5}\left(\theta_{5b}\right)}$.

The equations describing the end-effector orientation in terms of the angular coordinates are:

(11) $$g_{out}=e^{{\hat{\xi }}_{1}\theta_{1}}e^{{\hat{\xi }}_{2}\theta_{2}}e^{{\hat{\xi }}_{3}\theta_{3}}e^{{\hat{\xi }}_{4}-\theta_{3}}e^{{\hat{\xi }}_{5}\theta_{5}\left(\theta_{5b}\right)}g_{out0},$$

where the initial configuration of the handle is

(12) $$g_{out0}={\left[\begin{array}{cc} R_{out0} & p_{out0}\\ 0 & 1 \end{array}\right]}_{\left(4\times 4\right)},$$

(13) $$R_{out0}=\left[\begin{array}{ccc} \cos\left(\frac{\pi }{2}-\gamma \right) & -\sin\left(\frac{\pi }{2}-\gamma \right) & 0\\ \sin\left(\frac{\pi }{2}-\gamma \right) & \cos\left(\frac{\pi }{2}-\gamma \right) & 0\\ 0 & 0 & 1 \end{array}\right],$$

(14) $$p_{out0}=\left[\begin{array}{c} 218.4241\\ 722.6783\\ -141.0000 \end{array}\right],$$

where $R_{out0}$ is the rotation matrix of the handle, $p_{out0}$ represents the handle position coordinates in the global coordinate system, and γ = 0.8816 rad is the constant angle of the four-bar linkage.

The coordinates of the points on axes of the revolute joints (mm) are

(15) $$q_{1}=\left[\begin{array}{c} 0\\ 0\\ 0 \end{array}\right];q_{2}=\left[\begin{array}{c} -120\\ 0\\ 0 \end{array}\right];q_{3}=\left[\begin{array}{c} -120\\ 41\\ -151 \end{array}\right];\;q_{4}=\left[\begin{array}{c} -120\\ 356\\ -151 \end{array}\right];q_{5}=\left[\begin{array}{c} 30\\ 456\\ -261 \end{array}\right];q_{5b}=\left[\begin{array}{c} 30\\ 406\\ -261 \end{array}\right].$$

The axes of the revolute joints are given by the unit vectors

(16) $$\omega_{1}=\left[\begin{array}{c} 0\\ 0\\ 1 \end{array}\right];\omega_{2}=\left[\begin{array}{c} 0\\ 0\\ 1 \end{array}\right];\omega_{3}=\left[\begin{array}{c} -1\\ 0\\ 0 \end{array}\right];\omega_{4}=\left[\begin{array}{c} -1\\ 0\\ 0 \end{array}\right];\omega_{5}=\left[\begin{array}{c} 0\\ 0\\ 1 \end{array}\right];\omega_{5b}=\left[\begin{array}{c} 0\\ 0\\ 1 \end{array}\right].$$

**Figure 1:**
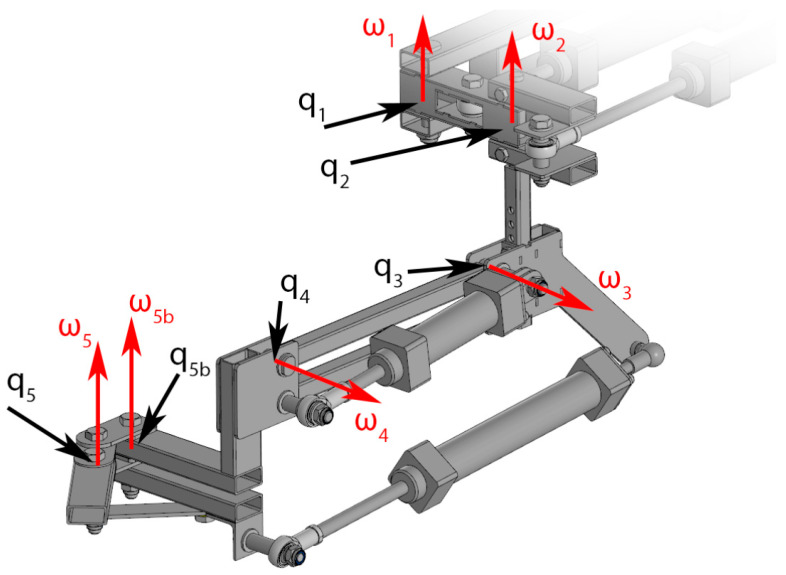
Components of the revolute joint twists.

From these components the twists vectors $\xi_{i}$ are formed according to Equations (2)–(4). The directions of the revolute joint axes shown in Figure 1.

**Figure 2:**
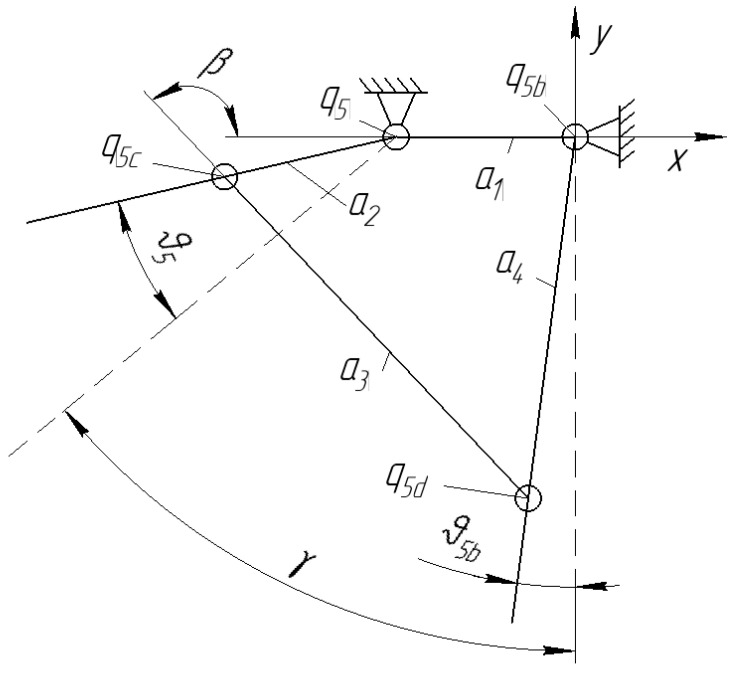
Schematic of the four-bar linkage mechanism.

To obtain the relation $\theta_{5}\left(\theta_{5b}\right)$, the projection of the four-bar linkage is considered separately and a local coordinate system is introduced. The schematic of the four-bar linkage is shown in Figure 2. The link lengths $a_{1},a_{2},a_{3},a_{4}$ are 50, 40, 110, and 100 mm, respectively.

The relationships in the four-bar linkage are derived using the vector loop-closure method. The complete system of loop equations is

(17) $$\left\{\begin{array}{c} a_{4}\cos\left(-\frac{\pi }{2}+\theta_{5b}\right)+a_{3}\cos\left(\beta \right)=a_{1}+a_{2}\cos\left(-\frac{\pi }{2}-\gamma +\theta_{5}\right)\\ a_{4}\sin\left(-\frac{\pi }{2}+\theta_{5b}\right)+a_{3}\sin\left(\beta \right)=a_{2}\sin\left(-\frac{\pi }{2}-\gamma +\theta_{5}\right) \end{array}\right.,$$

where β is the angle between link $a_{3}$ and the x-axis of the local frame. Squaring both equations, adding them, substituting the link lengths, and simplifying yields

(18) $$5\sin\left(\theta_{5b}\right)-4\cos\left(\theta_{5b}-\theta_{5}+\gamma \right)-2\sin\left(\theta_{5}-\gamma \right)+1=0.$$

Rewriting this in the form $A\cos\left(\theta_{5b}\right)+B\sin\left(\theta_{5b}\right)=C$ gives

(19) $$-4\cos\left(\theta_{5}-\gamma \right)\cos\left(\theta_{5b}\right)+\left(5-4\sin\left(\theta_{5}-\gamma \right)\right)\sin\left(\theta_{5b}\right)=2\sin\left(\theta_{5}-\gamma \right)-1.$$

Find the dependence $\theta_{5b}\left(\theta_{5}\right):$

(20) $$\theta_{5b}=\mathrm{atan}2\left(B,A\right)\pm \arccos\left(\frac{C}{\sqrt{A^{2}+B^{2}}}\right),\;\;A=-4\cos\left(\theta_{5}-\gamma \right);B=5-4\sin\left(\theta_{5}-\gamma \right);\;C=2\sin\left(\theta_{5}-\gamma \right)-1,\;\;\theta_{5b}\left(\theta_{5}\right)=\mathrm{atan}2\left(5-4\sin\left(\theta_{5}-\gamma \right),-4\cos\left(\theta_{5}-\gamma \right)\right)-\arccos\left(\frac{2\sin\left(\theta_{5}-\gamma \right)-1}{\sqrt{41-40\sin\left(\theta_{5}-\gamma \right)}}\right).\;$$

The sign before the arccosine is determined by the chosen assembly of the four-bar linkage. Within the working range of $\theta_{5b}$ the argument of the arccosine takes values from −0.3333 to −0.1540, whose absolute values are less than 1; hence the four-bar linkage singularities outside the workspace.

The inverse relation $\theta_{5}\left(\theta_{5b}\right)$ is obtained similarly. Introducing the shorthand $\chi =\theta_{5}-\gamma$,

(21) $$\chi =\mathrm{atan}2\left(B^{\prime },A^{\prime }\right)\pm \arccos\left(\frac{C\prime }{\sqrt{A\prime^{2}+B\prime^{2}}}\right)\;,\;\;A^{\prime }=4\cos\left(\theta_{5b}\right);B^{\prime }=4\sin\left(\theta_{5b}\right)+2;C^{\prime }=5\sin\left(\theta_{5b}\right)+1.$$

Returning to $\theta_{5}=\chi +\gamma$ yields

(22) $$\theta_{5}\left(\theta_{5b}\right)=\gamma +\mathrm{atan}2\left(4\sin\left(\theta_{5b}\right)+2,\;4\cos\left(\theta_{5b}\right)\right)-\arccos\left(\frac{5\sin\left(\theta_{5b}\right)+1}{\sqrt{20+16\sin\left(\theta_{5b}\right)}}\right).\;$$

In the operational region the argument of the arccosine here also lies within (−1, 1), reaching values from −0.1863 to 0.6614.

The mapping from the linear coordinates to the angular coordinates, $x_{pi}\to \theta_{i}$, which is part of the forward kinematics, is derived by considering the corresponding projections and establishing dependencies between geometric parameters and angles. However, it is not used in this study, which focuses on workspace verification.

Next, the inverse kinematics problem for the joint angles is solved: given the required coordinates of the handle $x_{des},\;y_{des},\;z_{des}$ and its rotation $\varphi_{des}$ about an axis parallel to the global z-axis, the necessary joint angles are determined.

Although the product of exponentials (PoE) formalism, combined with Paden–Kahan subproblems, is capable of yielding closed-form solutions, the specific geometry of the exoskeleton allows an even simpler approach: by projecting the spatial task onto a plane, the mechanism reduces to a classical planar three-link manipulator, for which a straightforward analytical solution exists.

It is observed that the angle $\theta_{3}$ is entirely determined by the required height coordinate of the end-effector $z_{des}$:

(23) $$\theta_{3}=-\arcsin\left(\frac{z_{des}-p_{outz0}}{c_{2}}\right),$$

where $c_{2}$ = 315 mm.

**Figure 3:**
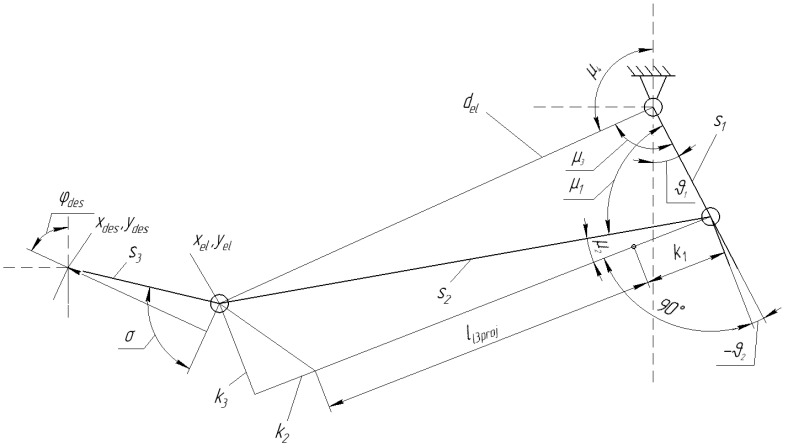
Schematic of the manipulator projection onto the horizontal plane.

Given that $\theta_{3}$ is known, the projection of the manipulator onto the XY plane is considered (Figure 3).

The problem reduces to finding the angles of a three-link planar manipulator. First, the coordinates of the “elbow” point (the axis of the revolute joint connecting the second and third links of the planar projective manipulator)—are found:

(24) $$x_{el}=x_{des}+s_{3}\sin\left(\varphi_{des}-\sigma \right),\;\;y_{el}=y_{des}-s_{3}\cos\left(\varphi_{des}-\sigma \right),\;$$

where σ = 1.3042 rad and $s_{3}$ = 326.5287 mm.

The distance from the origin to this point is

(25) $$d_{el}=\sqrt{x_{el}^{2}+y_{el}^{2}}$$

The projection length of the parallelogram mechanism is

(26) $$l_{l3proj}=c_{2}\cos\left(\theta_{3}\right)$$

The length of the second link of the projective planar manipulator is then

(27) $$s_{2}=\sqrt{{\left(l_{l3proj}+k_{1}+k_{2}\right)}^{2}+k_{3}^{2}},$$

with $k_{1}$ = 41 mm, $k_{2}$ = 100 mm, and $k_{3}$ = 150 mm.

The angles $\theta_{1}$ and $\theta_{2}$ are determined as

(28) $$\theta_{1}=\mu_{3}+\mu_{4}-\pi ,\;\;\mu_{3}=\arccos\left(\frac{s_{1}^{2}+d_{el}^{2}-s_{2}^{2}}{2s_{1}d_{el}}\right),\;\mu_{4}=\mathrm{atan}2\left(y_{el},x_{el}\right).$$

(29) $$\theta_{2}=\mu_{1}+\mu_{2}-\frac{\pi }{2},\;\;\mu_{1}=\arccos\left(\frac{s_{1}^{2}+s_{2}^{2}-d_{el}^{2}}{2s_{1}s_{2}}\right),\;\mu_{2}=\arcsin\left(\frac{k_{3}}{s_{2}}\right).$$

where $s_{1}$ = 120 mm.

Finally, the angle $\theta_{5}$ is found:

(30) $$\theta_{5}=\varphi_{des}-\theta_{1}-\theta_{2}-\frac{\pi }{2}+\gamma .$$

The angle $\theta_{5b}$ is then obtained from (20). The set of values $\theta_{1}$, $\theta_{2}$, $\theta_{3}$, $\theta_{5b}$ fully defines the manipulator configuration.

The mapping $\theta_{i}\to x_{pi}$ is obtained using the product of exponentials. From the joint angles $\theta_{i}$ the coordinates of the front ($p_{fi}$) and rear ($p_{bi}$) attachment points of each pneumatic cylinder are determined. Unlike the complete kinematic chain that yields the handle position, the product for an attachment point includes only the screws of those joints that lie between the base and the link that carries that point. The linear coordinate $x_{pi}$ is then computed as the distance between the front and rear attachment points. Note that when computing point coordinates with the PoE method, homogeneous coordinates (4 × 1 vectors) are used, whereas the distance calculation employs only the first three components of the resulting vector.

Thus, for each pneumatic cylinder we have

(31) $$x_{pi}=\left\|p_{fi_{\left(3\times 1\right)}}-p_{bi_{\left(3\times 1\right)}}\right\|.$$

where the front attachment points in the current configuration are expressed as

(32) $$p_{f1}=e^{{\hat{\xi }}_{1}\theta_{1}}p_{f10},\;\;p_{f2}=e^{{\hat{\xi }}_{1}\theta_{1}}e^{{\hat{\xi }}_{2}\theta_{2}}p_{f20},\;\;p_{f3}=e^{{\hat{\xi }}_{1}\theta_{1}}e^{{\hat{\xi }}_{2}\theta_{2}}e^{{\hat{\xi }}_{3}\theta_{3}}e^{{\hat{\xi }}_{4}-\theta_{3}}p_{f30},\;\;p_{f4}=e^{{\hat{\xi }}_{1}\theta_{1}}e^{{\hat{\xi }}_{2}\theta_{2}}e^{{\hat{\xi }}_{3}\theta_{3}}e^{{\hat{\xi }}_{4}-\theta_{3}}e^{{\hat{\xi }}_{5b}\theta_{5b}\left(\theta_{5}\right)}p_{f40},$$

and the rear attachment points are

(33) $$p_{b1}=p_{b10},\;\;p_{b2}=p_{b20},\;\;p_{b3}=e^{{\hat{\xi }}_{1}\theta_{1}}e^{{\hat{\xi }}_{2}\theta_{2}}p_{b30},\;\;p_{b4}=e^{{\hat{\xi }}_{1}\theta_{1}}e^{{\hat{\xi }}_{2}\theta_{2}}p_{b40},$$

The homogeneous coordinates of the front ($p_{fi0}$) and rear ($p_{bi0}$) attachment points in the initial configuration are

(34) $$p_{f10}=\left[\begin{array}{c} -60\\ 0\\ 0\\ 1 \end{array}\right];p_{b10}=\left[\begin{array}{c} -54\\ -405\\ 0\\ 1 \end{array}\right];\;\;p_{f20}=\left[\begin{array}{c} -190\\ 40\\ 0\\ 1 \end{array}\right];p_{b20}=\left[\begin{array}{c} -160\\ -617\\ 0\\ 1 \end{array}\right];\;\;p_{f30}=\left[\begin{array}{c} -170\\ 356\\ -191\\ 1 \end{array}\right];p_{b30}=\left[\begin{array}{c} -170\\ 41\\ -151\\ 1 \end{array}\right];\;\;p_{f40}=\left[\begin{array}{c} -175.5\\ 406\\ -325\\ 1 \end{array}\right];p_{b40}=\left[\begin{array}{c} -157\\ -154\\ -291\\ 1 \end{array}\right].$$

After substitution and simplification, one obtains the explicit expressions for the linear coordinates as functions of the angular coordinates:

(35) $$x_{p1}=\sqrt{170541-6480\cos\left(\theta_{1}\right)-48600\sin\left(\theta_{1}\right)},$$

(36) $$x_{p2}=\sqrt{\psi_{1}+\psi_{2}},\;\;\psi_{1}={\left(120\cos\left(\theta_{1}\right)+10\sqrt{65}\cos\left(\theta_{1}+\theta_{2}-\;\mathrm{atan}\left(\frac{4}{7}\right)\right)-160\right)}^{2},\;\;\psi_{2}={\left(120\sin\left(\theta_{1}\right)+10\sqrt{65}\sin\left(\theta_{1}+\theta_{2}-\;\mathrm{atan}\left(\frac{4}{7}\right)\right)-617\right)}^{2},$$

(37) $$x_{p3}=\sqrt{100825+25200\sin\left(\theta_{3}\right)},$$

(38) $$x_{p4}=\sqrt{\eta_{1}+\eta_{2}+\eta_{3}\;},\;\eta_{1}={\left(315\cos\left(\theta_{3}\right)-205.5\sin\left(\theta_{5b}\right)+245\right)}^{2},\;\eta_{2}={\left(205.5\cos\left(\theta_{5b}\right)-187\right)}^{2},\;\;\eta_{3}={\left(315\sin\left(\theta_{3}\right)+34\right)}^{2}.$$

### 3.3. Data Processing Algorithm for Workspace Definition

Since previous research stages demonstrated that mathematical modelling cannot fully predict all potential collisions between exoskeleton links, additional verifications are required. A three-stage algorithm is proposed for this purpose.

In the first stage, a discrete 3D cloud of target manipulator handle positions is generated. Inverse kinematics (IK) calculations are performed for each position to verify the entire exoskeleton configuration. The generated cloud constitutes a uniform coordinate grid within a sphere of 1-metre radius, centred at the structural point $Q_{0}$ (the attachment point of the exoskeleton to the user’s shoulder joint), with a spatial step ΔQ. For each spatial point, the handle rotation angle φ around its longitudinal axis is specified within a range of $\varphi \in [\varphi_{min},\;\varphi_{max}]$ with an angular step Δφ. Consequently, the total set of configurations to be verified is the Cartesian product of the spatial coordinates and the discrete values of φ. For each configuration $\left(x,\;y,\;z,\varphi \right)$, the solution of the inverse kinematics problem is calculated. The IK model is implemented by porting the analytical equations (Section 3.2), verified in MATLAB, into C# code.

Ensuring compatibility between the MATLAB kinematic model and the Unity (C#) simulation environment is a critical step. MATLAB operates in a right-handed Cartesian coordinate system by default, whereas the Unity physics engine uses a left-handed system. Direct formula porting resulted in a mirrored workspace and inverted Euler angles. This was resolved by developing a software adapter performing affine transformations on input data: the X-coordinate is sign-inverted, and the Y and Z axes are swapped. Additionally, a correction parameter $\varphi_{offset}$ was introduced to align the zero positions of the handle rotation axis.

To achieve high performance via the C# Job System, an algorithm was required that operates without dynamic memory allocation, utilizing value types exclusively. The mathematical logic was rewritten using structures, and configuration arrays were replaced with NativeArray unmanaged buffers. This eliminated garbage collector (GC) calls during cycles comprising millions of iterations. During the translation of inverse trigonometric functions (arcsine, arccosine) from MATLAB to C#, floating-point precision loss issues were identified. At the kinematic boundaries, function arguments could microscopically exceed the admissible range, leading to NaN returns and corrupted calculations. To mitigate this, a filter was implemented to strictly clamp operands to mathematically valid limits before passing them to trigonometric functions.

The second stage is executed directly within parallel Job System tasks and does not require interaction with the engine’s physics subsystem. Each manipulator position from Stage 1 is checked against two groups of constraints. First, the existence of a real-valued IK solution for the piston lengths $x_{p1}$, $x_{p2}$, $x_{p3}$, $x_{p4}$ is verified and compared against the structural limits $\left[L_{min}(i),L_{max}(i)\right]$. Second, the angular coordinates $\theta_{1},\theta_{2},$ and $\theta_{5b}$ are verified against anatomical and structural limit constraints.

In the third stage, only configurations that successfully passed the mathematical verification are processed. For each configuration, the following procedure is executed: the coordinates of key manipulator nodes and the angle φ, calculated via inverse kinematics, are applied to the components of the virtual 3D model. This updates the positions and spatial orientations of all links in the kinematic chain. Once all objects are transformed, an update of the internal Bounding Volume Hierarchies (BVH) of the PhysX engine (NVIDIA, Santa Clara, CA, USA) is triggered. Subsequently, pairwise geometric intersection checks are performed between the structure element colliders. Additionally, target point reaching accuracy is verified by calculating the Euclidean distance between the specified configuration and the actual position of the end-effector.

Because Stage 3 performs instantaneous geometric intersection queries without invoking the LCP solver, it deliberately disregards friction and contact forces. However, for a static reachability analysis, only geometric overlap is relevant: any collision-free configuration is kinematically attainable provided sufficient actuator torque is available. The dynamic feasibility, including friction-induced binding, will be assessed in the subsequent control-oriented validation.

## 4. Results

### 4.1. Hardware Description of the Upper Limb Exoskeleton

The manipulator design in this study is based on an architecture similar to the Pneu-WREX system [5], as it provides the necessary degrees of freedom (DoF) for replicating upper limb movements (flexion/extension in the shoulder and elbow joints, abduction/adduction, and rotation). However, the original design was substantially re-engineered to account for manufacturing constraints and to reduce the cost of prototype fabrication. This was achieved by simplifying individual assemblies, substituting materials, and standardising components. Utilising a validated architecture allows research efforts to be directed away from mechanical design towards resolving the primary scientific challenge: the development of an effective control system adapted to the specificities of the rehabilitation process.

**Figure 4:**
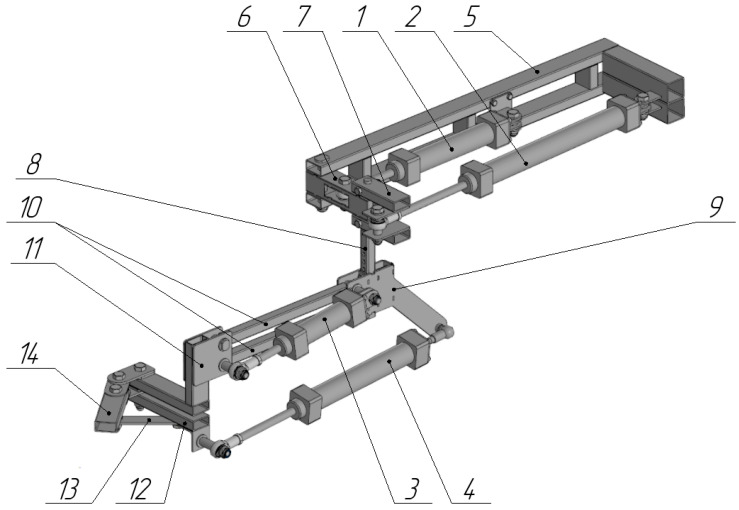
Three-dimensional model of the upper limb exoskeleton: 1–4—pneumatic cylinders; 5—base; 6—shoulder link; 7—shoulder lever; 8—axis; 9— bracket; 10—parallelogram mechanism links; 11–14—four-bar linkage.

The overall appearance and structural components of the exoskeleton manipulator are shown in Figure 4 (the handle attachment is omitted).

The primary structural elements of the exoskeleton are fabricated from grade St3 steel (analogous to S235JR/A36), using 20 × 40 mm and 20 × 20 mm profile tubes and laser-cut sheet metal parts. This material choice provides the necessary rigidity and durability at moderate cost. Consequently, even under the maximum operating pressure of 9 bar and the full weight of a human arm, the anticipated elastic deflections are negligible and do not compromise the geometric accuracy of the workspace verification. In addition, the simulation framework supports optional scaling of the collision meshes to introduce a conservative safety margin; this buffer zone can be activated to ensure that the real manipulator remains within the verified safe workspace even under worst-case combinations of load, manufacturing tolerances, and assembly misalignments.

Pneumatic cylinders 1 and 2 are attached to the base 5 via joints. Cylinder 1 defines the displacement of the shoulder joint axis in the axial plane. Cylinder 2 drives the shoulder lever 7, which is connected to the bracket 9 via axis 8, rotating the shoulder in the axial plane. The implementation of two degrees of freedom in this assembly is necessitated by the requirement to emulate complex shoulder joint kinetics.

Axis 8 features adjustment holes for varying the bracket 9 height within a range of 106 mm to 151 mm in 15 mm increments. The present investigation uses setting of 151 mm. Cylinder 3 drives the parallelogram mechanism, raising and lowering link 11 of the four-bar linkage such that it remains parallel to bracket 9, thereby rotating the shoulder in the parasagittal plane. Cylinder 4 actuates the four-bar linkage.

The patient handle is attached to the output link 14 of the four-bar linkage. The use of a four-bar linkage allows cylinder 4 to be positioned so that it does not obstruct movement. Cylinder 4 is mounted using spherical bearings—specifically, an angular joint and a rod-end bearing for the rear and front attachment points, respectively. The use of spherical bearings facilitates concurrent movements of cylinders 3 and 4. Cylinders 1–4 have piston diameters of 40 mm and stroke lengths of 150, 300, 75, and 200 mm, respectively. The operating pressure ranges from 1 to 9 atmospheres.

**Figure 5:**
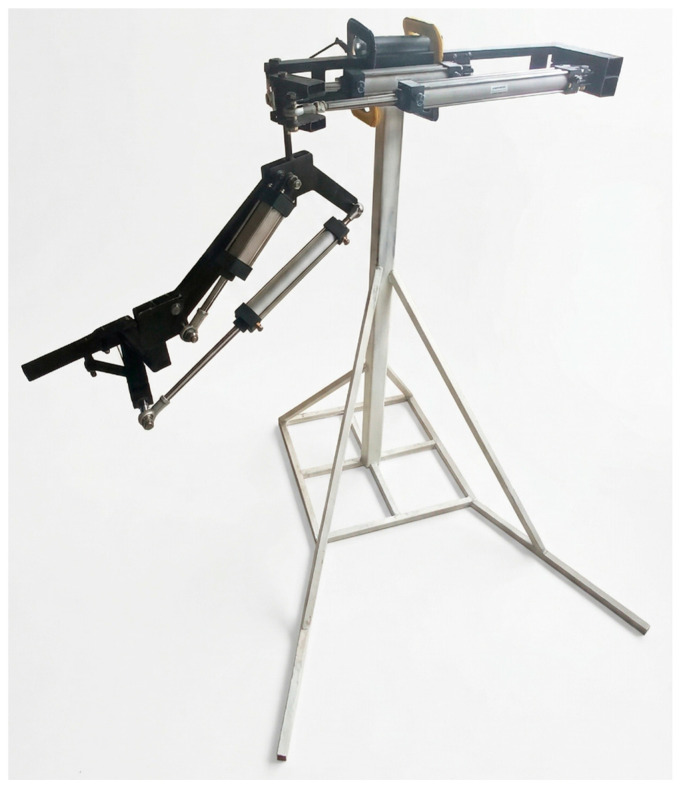
Hardware implementation of the upper limb exoskeleton.

The implemented physical model of the exoskeleton (exemplified by the left-limb configuration) is presented in Figure 5. The pneumatic cylinders are driven by proportional pressure regulators connected to a central compressed air supply. Closed loop pressure control is performed by the low-level controller, which receives setpoints from the kinematic trajectory planner. The detailed design of the force pressure control loop will be the focus of subsequent work.

The simulations were conducted on the following hardware:

- CPU: AMD Ryzen 9 5950X (16 physical cores, 32 logical threads, base clock 3.4 GHz);
- GPU: NVIDIA GeForce RTX 4080 16 GB;
- RAM: 128 GB DDR4.

### 4.2. Software Description

MATLAB R2020b was utilised for kinematic model development and preliminary verification. To implement the data processing algorithm described in Section 3.2 and to provide a visual representation of the results, a specialised software suite was developed using the Unity engine (version 6000.3.5f1). The developed software functions as a digital twin of the exoskeleton, enabling the analysis of the manipulator’s structural constraints.

The primary objective of the simulation environment is to identify and register physically incorrect mechanism states that are difficult or impossible to detect using analytical methods alone. The software visualises the exoskeleton state for every mathematically reachable point and checks for design errors. These errors include geometric collisions of components, kinematic chain disjunctions, and the inability of the manipulator to reach specified Cartesian coordinates. Examples of the software suite’s operation in identifying these error types are presented below.

During the third stage of the algorithm (Section 3.2), the software positions the 3D models of all manipulator links in virtual space according to the computed inverse kinematics. The combination of the mathematical model and the three-dimensional simulation environment makes it possible to detect and systematically eliminate various special configurations that are inadmissible for safe operation. The following figures illustrate the types of problems that can arise if the workspace is not rigorously verified.

**Figure 6:**
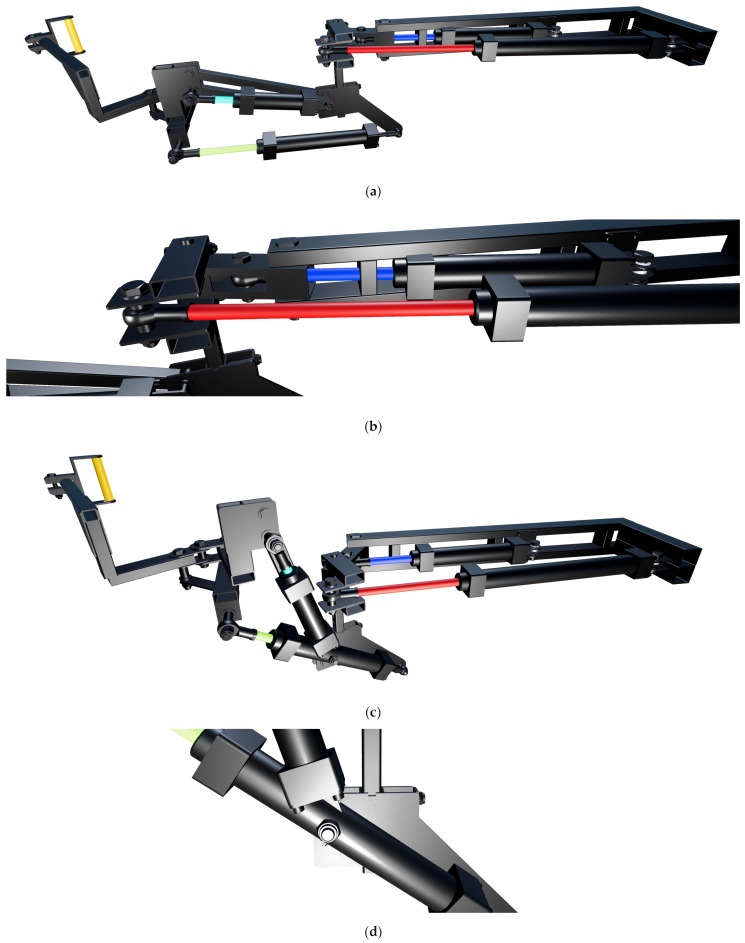
Collision examples: (**a**,**c**) overall view of the exoskeleton; (**b**,**d**) enlarged details of the colliding meshes.

Figure 6 shows a configuration in which the handle position formally satisfies the analytical constraints, yet the solid-state meshes intersect. The simulator’s physics engine identifies the penetration between the pneumatic cylinder housing and the supporting frame. This example visualises a collision that would lead to mechanical jamming or structural failure in a physical prototype.

**Figure 7:**
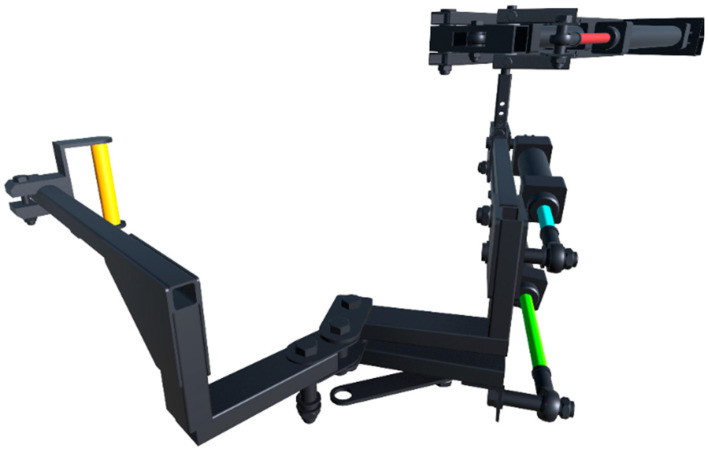
Example of a kinematic disjunction (the swivel mounts are separated, making assembly impossible).

A second type of inadmissible configuration is presented in Figure 7. Owing to the hybrid kinematics, the inverse kinematics may return a formally existing but physically unrealisable solution. The software visualises this as a spatial disjunction between the swivel mounts, confirming that the assembly and operation of the mechanism in this configuration are impossible without joint destruction.

**Figure 8:**
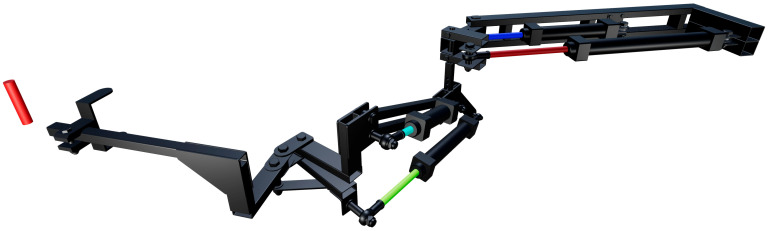
Positioning error (the handle cannot reach the target point within the allowed tolerance).

Figure 8 illustrates a third critical scenario: the end-effector fails to reach the required target point even though all generalised coordinates, piston lengths, and joint angles lie within their mathematical tolerances. The interface displays the commanded cloud point together with the actual handle position, and the Euclidean distance between them exceeds the specified threshold, flagging the configuration as functionally inadmissible.

Thus, the developed software suite not only identifies physically incorrect states, but also enables their visual inspection and systematic elimination. Together with the analytical PoE model, the three-dimensional simulation ensures that every configuration admitted to the final safe workspace matrix is free of geometric collisions, kinematic disjunctions, and positioning errors.

Physical interactions were computed by the integrated NVIDIA PhysX 4.1.2 engine (Unity version 6000.3.5f1). The mathematical model was compiled with the Unity Burst Compiler, and the parallelization framework relied on the Unity C# Job System. The standard asynchronous frame-based simulation (Auto-Simulation/FixedUpdate) was completely disabled to retain strict control over computational timing. Collision detection was performed via direct synchronous penetration vector calculation using Physics. ComputePenetration(), preceded by an explicit Physics. SyncTransforms() call before each verification iteration to manually update the BVH tree. The default contact offset was kept at the PhysX default value of 0.01 m (10 mm).

The physical model contained three Mesh Colliders representing the housings of the first, third, and fourth pneumatic cylinders (2724, 3168, and 1168 triangles, respectively). The housing of the second cylinder was excluded from the collision model because its fixed location makes geometric interference structurally impossible; only the Capsule Collider of its moving piston rod was retained. In total, four Capsule Colliders were employed for the piston rods and four Box Colliders for the frame elements. The overall mesh complexity processed by the PhysX engine was below 8000 triangles.

**Figure 9:**
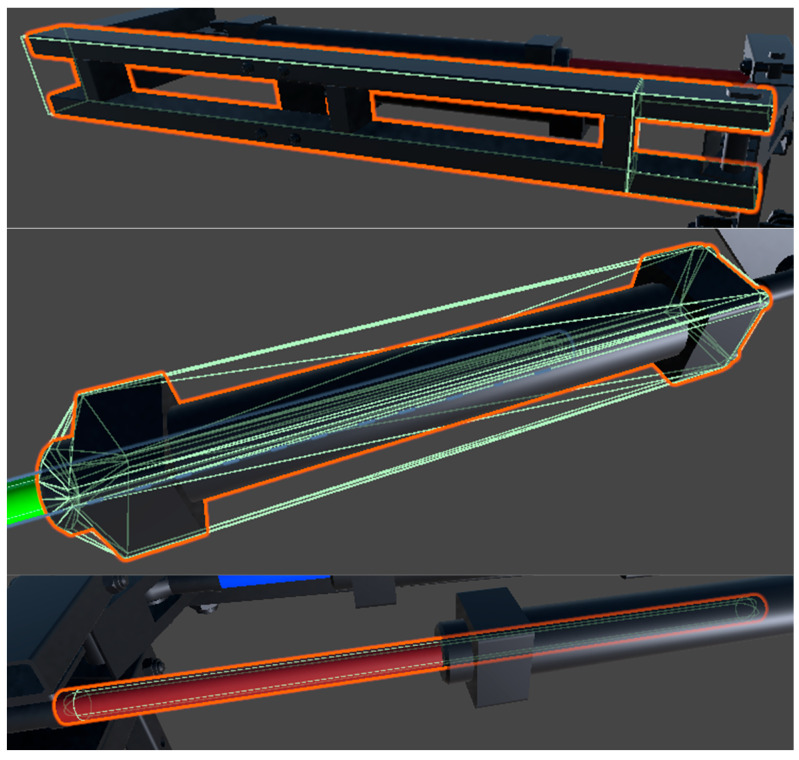
Examples of the collider setup.

No additional safety-margin scaling or artificial buffer zones were applied; collision registration relied exclusively on the actual geometric boundaries of the assigned colliders. The architecture nevertheless inherently supports the optional introduction of user-definable safety margins (e.g., scaling colliders or offsetting mesh vertices) if required for production preparation. Figure 9 illustrates examples of the collider setup.

The current simulation employs the nominal CAD-derived collision meshes without an explicit buffer zone. Nevertheless, the architecture inherently supports the application of user-definable safety margins (e.g., scaling colliders or offsetting mesh vertices), which can be activated during production preparation to account for assembly tolerances.

Pneumatic tubes and sensor cables are routed along the rigid links and secured by cable ties; they do not protrude into the inter link gaps. Consequently, they present no additional snagging risk and were omitted from the collision model. If needed in future design variants, simplified flexible colliders can be added without modifying the verification algorithm.

### 4.3. Simulation Results

In accordance with the proposed algorithm, a discrete 3D point cloud of target manipulator handle positions was generated during the first stage. The parameters (radius, step, angles, etc.) for the analysed region are specified in Table 2.

During the second stage, the specified points were verified against the kinematic model constraints. This stage involved checking two primary conditions:

1. The existence of a real-valued inverse kinematics solution for the piston lengths corresponding to the analysed point, and ensuring these lengths comply with the limits specified in Table 3 ($x_{p1}$, $x_{p2}$, $x_{p3}$, $x_{p4}$).
2. Verification of the angular coordinates $\theta_{1},\theta_{3},$ and $\theta_{5b}$ against the anatomical and structural constraints, also presented in Table 3.

The results (Table 4) demonstrate that mathematical filtering in Stage 2 rejects 99.60% of configurations before invoking the computationally expensive physical intersection check functions. This provides a key justification for the three-stage architecture of the algorithm.

Only configurations that successfully passed the mathematical verification are transferred to the third stage. For each configuration, the following procedure is executed: the coordinates of the key manipulator nodes and the angle φ, calculated via inverse kinematics, are applied to the components of the virtual 3D model. Consequently, the positions and spatial orientations of all links in the kinematic chain are updated. Following the transformation of all objects, an update of the internal Bounding Volume Hierarchies of the PhysX engine is triggered. Subsequently, pairwise geometric intersection checks are performed between the structural element colliders. Additionally, target point accuracy is verified by calculating the Euclidean distance between the specified configuration and the actual position of the end-effector.

Performance evaluation of the algorithm showed that generating the initial dataset (757 million configurations) required 702.7 s (exceeding one million values per second). Inverse kinematics calculations and mathematical filtering using the Unity Burst Compiler were completed in just 33.4 s (at a rate exceeding 22 million operations per second). Since the mathematical filter rejected 99.6% of invalid configurations prior to engaging the physics engine, the final check for resource-intensive physical collisions took only 376.5 s. Thus, without the integrated parallelised mathematical filter, collision detection for the entire dataset would have exceeded 26 h. The proposed pipelined architecture reduced the total workspace verification time to approximately 20 min.

Table 5 provides representative values for several key angular positions, allowing for an assessment of the percentage of valid points for each specific angle.

.

**Figure 10:**
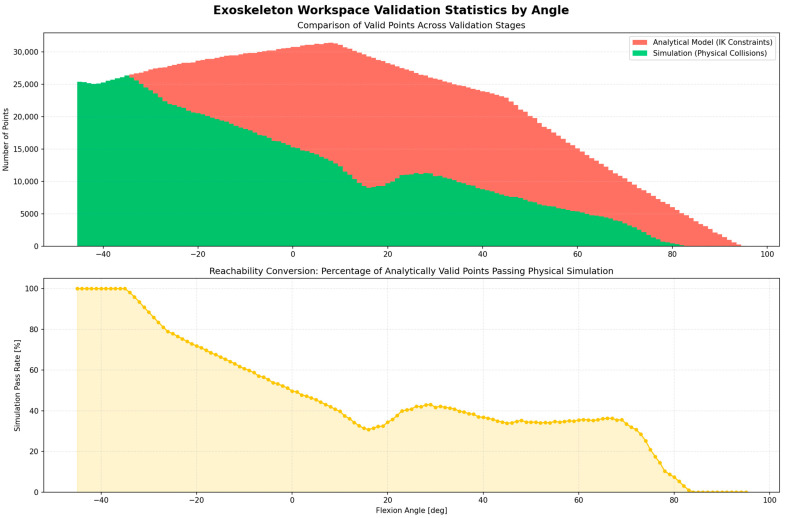
Comparative histogram of valid points after Stage 2 (mathematical constraints) and Stage 3 (physical collisions).

In the course of analysing the algorithm’s output, the overall efficiency for each angle was evaluated, and a histogram was constructed showing the number of permissible points at each angle, considering both mathematical model and physical collision checks. The results of the comparative analysis are presented in Figure 10.

Analysis of the histogram (Figure 10) demonstrates that verifying points solely against mathematical model constraints (Stage 2) yields an overestimation of the workspace volume. Angles φ exceeding 85–90 degrees are inapplicable for operation. The implementation of the physical collision detection algorithm (Stage 3), which accounts for impacts between frame elements and cylinder rods, results in a significant reduction in valid points. This decrease (exceeding 80% at most angles) confirms that in the design of hybrid kinematic mechanisms with pneumatic drive, physical link intersections—rather than stroke length limits—serve as the primary limiting factor for system manoeuvrability. Ignoring this factor during mathematical modelling would inevitably lead to mechanism jamming under real-world conditions.

**Figure 11:**
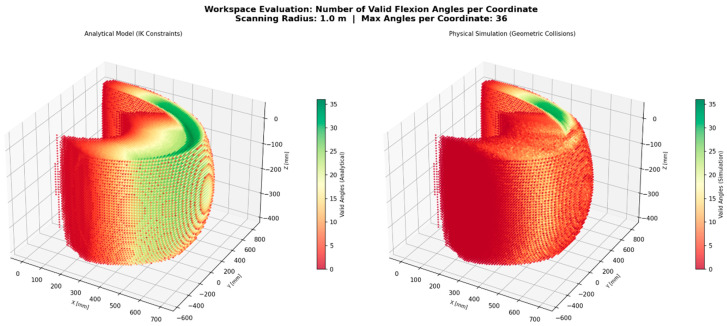
Distribution of the manoeuvrability coefficient in 3D space (red—low manoeuvrability, green—high).

To qualitatively evaluate the distribution of degrees of freedom within the reachable workspace, a 3D visualisation was constructed, reflecting the integral manoeuvrability coefficient at each point (Figure 11). This coefficient indicates the proportion of valid configurations (at various end-effector orientation angles φ) that are physically realisable at a given coordinate (x,y,z).

Visual analysis of the gradient (Figure 10) allows for several critical conclusions regarding the ergonomics of the developed design:

- The valid point cloud exhibits pronounced asymmetry in the workspace. The exoskeleton mechanism is designed to operate primarily in the user’s anterior hemisphere, aligning with the natural biomechanics of the shoulder joint during rehabilitation or daily activities.
- Zones with a high manoeuvrability coefficient (green markers) are concentrated in a compact central region. In this zone, the manipulator provides maximum end-effector orientation variability without reaching cylinder stroke limits.
- Peripheral regions (red and yellow markers) are characterised by a sharp decline in manoeuvrability. In these areas, the exoskeleton can reach the target point only at one or two strictly fixed joint orientations, necessitating increased attention when generating virtual trajectories in immersive environments to avoid forced mechanism stops.

**Figure 12:**
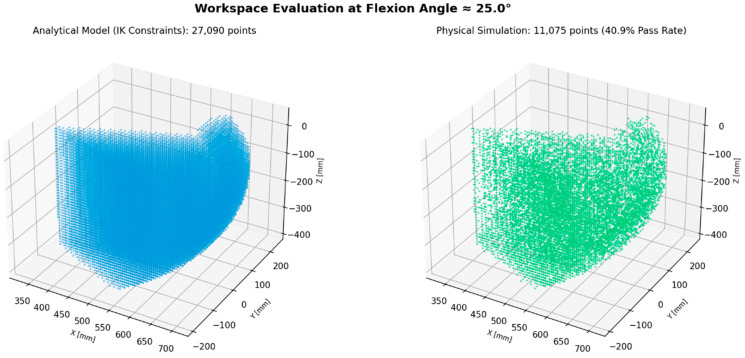
Density distribution of valid configurations in the XY plane at a fixed angle.

For a more detailed study of the manoeuvrability distribution profile, a planar cross-section of the workspace at a fixed angle was generated (Figure 12).

The plot confirms the presence of a «manoeuvrability core» with a high concentration of successful points within a narrow coordinate corridor. The gradient stratification from green to red as distance from the workspace centre of mass increases occurs non-linearly, which is characteristic of hybrid kinematic mechanisms with pneumatic drive.

The conducted analysis confirms the feasibility and effectiveness of the proposed algorithm. Its application enables the identification of additional inadmissible exoskeleton segment positions, which is of paramount importance for the safety of both the user and the system as a whole.

## 5. Discussion

### 5.1. Discussion of Results

The implementation of the proposed hybrid kinematic state verification algorithm has yielded an exhaustive safe workspace matrix for the pneumatic upper-limb exoskeleton. Analysis of how configurations progressed through different filtering stages (as detailed in Table 4) revealed several fundamental patterns critical for the design of such robotic systems.

Firstly, the results confirm the high computational efficiency of the analytical filter based on the PoE method. The mathematical rejection of configurations violating pneumatic cylinder rod stroke limits and anatomical angular constraints reduced the initial dataset of 758 million points by 99.6%. This demonstrates the rationality of employing preliminary analytical filtering to alleviate computational loads before initiating resource-intensive collision detection algorithms.

Secondly, a key scientific result is the quantitative assessment of the discrepancy between the theoretical (mathematical) and actual (physical) manipulator workspaces. As shown by the analysis in Stage 3, nearly half (approximately 1.4 million points) of the more than 3 million configurations deemed entirely correct from an analytical inverse kinematics standpoint were rejected by the physics engine due to geometric intersections of solid structural elements. This discrepancy empirically proves that for mechanisms featuring closed kinematic chains (including four-bar linkages and parallel pneumatic cylinders), classical analytical models are insufficient for ensuring user safety. Neglecting the spatial collision verification stage in a 3D environment would inevitably result in mechanical failure of the equipment when attempting to reach a formally permitted but physically obstructed coordinate.

Thirdly, topological analysis of the verified point cloud (Figure 11 and Figure 12) identified a pronounced asymmetry in the exoskeleton’s workspace. The resulting «manoeuvrability core» is shifted into the anterior hemisphere, fully satisfying biomechanical requirements for executing fundamental musculoskeletal rehabilitation patterns or typical daily activities. The observed density gradient of valid configurations imposes specific constraints on the development of virtual reality scenarios: interactive virtual objects must be spatially positioned exclusively within the identified zone. The manoeuvrability coefficient map inherently highlights regions of low orientational freedom; no large internal voids were observed, but the identified peripheral low-manoeuvrability zones must be treated as “trap states” during trajectory planning—the exoskeleton may enter them but could be forced to retract along a specific orientation path. The proposed safe matrix provides the necessary information to avoid such situations.

Fourthly, while the offline verification process leverages multi-threaded CPU optimizations (C# Job System, Burst Compiler) to achieve high throughput, the resulting safe workspace matrix is a compact, pre-computed lookup table that requires only a simple membership test. This separation between the computationally intensive offline stage and the lightweight online query makes the runtime safety filter fully portable to embedded controllers with modest computational resources, without any dependency on the original simulation environment.

Thus, the set of safe states obtained during this study will be applied in the development of the upper exoskeleton control system and will serve as the foundation for its safety subsystem.

### 5.2. Comparison with Existing Research

The validity of the proposed design and verification methodology for the pneumatic exoskeleton workspace is confirmed by a comparative analysis with existing studies in the field.

The two-component architecture proposed in this work aligns with contemporary trends in systems engineering design [20,21]. Comparative studies demonstrate the superiority of game engines (specifically Unity) over traditional environments (such as Gazebo) for tasks involving high-frequency discrete positioning and visualisation [22]. In contrast to classical analytical modelling based on D-H parameters in heavy CAD systems like SolidWorks [23], the application of Unity enables real-time interactive state verification. At the same time, the isolated use of graphics engines does not allow for the implementation of rigorous mathematical models of non-linear processes [24], necessitating a powerful analytical core (MATLAB) for calculating forward and inverse kinematics and filtering static forces [25,26].

A direct quantitative comparison of static collision-query throughput across all simulators listed in Table 1 is difficult, because published benchmarks focus on overall simulation fidelity rather than on time-agnostic intersection tests. Nevertheless, several architectural observations favour the chosen Unity-based pipeline. Platt and Ricks [18] reported that Gazebo’s physics step consumes a substantial fraction of the real-time budget even for modest scenes, whereas Unity achieves higher simulation update rates, indicating more efficient handling of spatial computations. Audonnet et al. [19] noted that Gazebo, Webots, and CoppeliaSim are inherently coupled to the dynamics time step, which limits their ability to perform isolated collision queries without overhead. Wijaya et al. [22] further demonstrated that Unity provides higher step rates than Gazebo in pick-and-place tasks requiring repeated collision checks.

Extrapolating from these findings and from the architectural analysis in Table 1, it is reasonable to expect that conventional LCP- or DAE-coupled simulators would be impractical for verifying 758 million static configurations. Our own measurements indicate that even the optimised PhysX stage required approximately 376 s for the 3 million analytically valid configurations; scaling a dynamics-coupled query to the full set would conservatively exceed 26 h. Recent reviews of upper-limb exoskeletons [1,2] emphasise the necessity of exhaustive geometric verification for closed-chain mechanisms, yet note the absence of dedicated tools for high-throughput static workspace analysis. The present work therefore addresses this gap, demonstrating not only that Unity is performant for dynamic tasks, but also that its ability to decouple collision detection from time integration uniquely qualifies it for massive static verification.

A critical aspect of verifying hundreds of millions of manipulator configurations is algorithmic performance. Research in [27] has proven the high computational efficiency of parallel collision detection for complex polygonal meshes within Unity. These results directly support the selection of a game engine for the third stage of the presented algorithm: offloading physical intersection checks to a multi-threaded subsystem (C# Job System) allows for the instantaneous rejection of invalid states without triggering an unnecessary dynamic simulation cycle, which is unattainable in monolithic physics engines.

It is worth emphasising that, although the verification pipeline was demonstrated on a specific pneumatic upper-limb exoskeleton, the underlying architecture is inherently modular. The analytical stage relies on the PoE formalism, which can be adapted to any kinematic chain by redefining the screw axes and the initial transformation matrices in Equations (9)–(38). The collision-detection stage only requires importing the CAD assembly of the target device into Unity and configuring the corresponding colliders. The filtering logic—rod-stroke limits, angular constraints, and mesh-intersection checks—remains unchanged. Consequently, the proposed method can be applied, without algorithmic modification, to other exoskeleton configurations, hybrid kinematic mechanisms, or even electrically actuated devices, provided that a valid kinematic model and a solid-state geometric model are available.

The development of a safe kinematic chain requires strict consideration of the anatomical limits of the human musculoskeletal system [28]. Study [29] emphasises the mandatory iterative assessment of workspace volume and the identification of singularities under worst-case loading scenarios. Our approach extends these methodologies: the analytical filtering of kinematic states followed by the verification of a manipulator’s digital twin [30,31] ensures the exclusion of physically unrealisable spatial positions, which is particularly critical for hybrid kinematics systems.

Clinical practice confirms that integrating robotic therapy with virtual reality provides a synergistic effect by stimulating neuroplasticity [32]. In this context, the creation of a digital twin [33] performs a dual function: it serves not only as a tool for workspace calculation during the design phase but also as the basis for an immersive environment where patients interact with virtual objects [34]. The set of admissible manipulator positions generated in this study provides the foundation for implementing safe Human–Robot Interaction (HRI) interfaces, ensuring that target objects in the virtual scene remain within the manipulator’s verified workspace.

### 5.3. Limitations

Despite the demonstrated effectiveness of the proposed hybrid kinematic verification algorithm, the current study has several objective limitations stemming from the assumptions made and the architecture of the computational experiment.

Firstly, the workspace analysis was conducted with a specified positioning step of 0.01 m and an angular step of 1°. While this approach ensures the reachability of isolated Cartesian coordinates, it does not account for the dynamic aspects of transitioning between adjacent configurations. It should be stressed that the validation presented in this study is entirely computational and CAD-based; no experimental measurements have yet been performed on the physical prototype. The target application—post-stroke upper-limb rehabilitation—is characterized by low-velocity, quasi-static movements where inertial effects are negligible, as evidenced by the foundational Pneu-WREX architecture on which our design is based [5]; consequently, dynamic momentum and boundary overshoot risks are deferred to future investigations. The influence of inertial forces, joint friction, and, most critically for the chosen hardware, the thermodynamic latencies of air compressibility in pneumatic cylinders requires separate investigation within the framework of full system dynamics modelling. Thermodynamic transients, valve switching delays and air compressibility are not represented in the current digital twin; the verified workspace matrix serves as a geometric safety envelope, and compensation of pneumatic nonlinearities is assigned to the lower-level force-pressure controller, consistent with the hierarchical control architecture discussed in Section 2.1. Joint friction and seal stiction primarily affect the actuation force required to reach a configuration rather than its geometric attainability; at low operating pressures, certain mathematically reachable points may become practically inaccessible—a matter belonging to the dynamic force-domain analysis. Regarding the discrete sampling, the spatial (0.01 m) and angular (1°) steps are substantially smaller than the characteristic link dimensions and inter-element clearances. The probability that a collision exists strictly between two sampled points while escaping detection at both is therefore negligible, and an adaptive mesh refinement can further increase resolution if needed. For continuous trajectory safety, any candidate path can be sampled at a resolution compatible with the grid step (0.01 m, 1°) and each intermediate point checked against the pre-computed safe set. The development of a continuous trajectory planner that formally guarantees collision-free motion using the safe workspace matrix is a natural extension of this work.

Secondly, the physical simulation implemented in Unity focuses exclusively on preventing internal geometric collisions between the rigid links of the metal structure and the pneumatic actuators. In the current version of the digital twin, the biomechanical model of the human upper limb is represented in a simplified form. Deformations of soft tissues and the non-linear displacement of the instantaneous center of rotation of the biological shoulder joint during arm abduction are not considered. In clinical practice, soft-tissue compliance may locally modify the collision boundary; this can be accommodated by introducing patient-specific safety offsets (e.g., a virtual padding layer) during the calibration phase without altering the verification algorithm itself.

Thirdly, the established constraints on the generalised coordinates (specifically the forearm rotation limits) are based on averaged normative anthropometric data. In clinical applications for the rehabilitation of patients with severe post-stroke hemiparesis, spasticity, or joint contractures, these limits may prove excessively broad. The framework supports individualisation by tightening the angular limits according to the patient’s measurements and re-running the verification pipeline; the present results, however, do not cover such personalised regimes.

Furthermore, this study does not address the development of a dynamic model for the upper exoskeleton manipulator. In accordance with [5], this is not a critical drawback at low velocities and when implementing additional corrective control algorithms, which will be the focus of further research.

Additional factors that lie beyond the current scope include: sensor noise and encoder resolution (no formal sensitivity analysis has been performed; a conservative margin, e.g., inflating collision meshes by the maximum expected positioning error, can be introduced as a practical interim solution); long-term mechanical wear, which may introduce slight axis misalignments mitigated by periodic recalibration and conservative collision margins; external obstacles (wheelchairs, therapy tables), which were not included in the virtual scene but can be added by importing the relevant geometry; and thermal effects on pneumatic pressure, which influence dynamic response rather than geometric bounds and will be addressed during the dynamic modelling phase. Finally, the validation is currently limited to analytical consistency checks between MATLAB and Unity, supplemented by CAD interference verification; experimental validation using a 3D motion-capture system with the physical prototype is planned as the next step.

Although a formal emergency protocol—immediate venting of the affected cylinder(s) followed by a controlled, low-pressure return to the home configuration—is foreseen in the safety architecture described in Section 2.1, its detailed implementation belongs to the control-system design and lies beyond the scope of the present kinematic verification.

The achievable positioning accuracy and repeatability of the pneumatic cylinders have not yet been experimentally characterised. Ongoing development of the control subsystem employs two electro-pneumatic converters driven with iterative step adjustment near the target to balance smooth positioning against settling time. Quantitative data on the positioning precision, as well as the performance of the final closed-loop controller, will be reported in a dedicated follow-up study.

Thus, these limitations do not diminish the validity of the results regarding the exoskeleton’s mechanics but rather define directions for future research. Subsequent work will aim to integrate dynamic pneumatic actuator models, personalise kinematic limits, implement sensor-aware safety margins, account for lifecycle wear, and realise a continuous trajectory planner. In summary, while the verified workspace constitutes a necessary condition for collision-free operation, it must be complemented by a dynamic safety layer that enforces velocity, acceleration, and force constraints during real-time control.

## 6. Conclusions

In this study, the task of defining and verifying the safe workspace of a pneumatic upper-limb exoskeleton intended for post-stroke upper-limb rehabilitation and immersive professional training (rescue services, industrial operators) was resolved. To overcome the architectural limitations of classical LCP simulators (Gazebo, Webots) and analytical environments (MATLAB Simscape), a hybrid algorithm was proposed and validated, combining PoE-based analytical filtering with high-performance collision detection in Unity.

The following primary results were obtained during the study:

- The limitations of isolated mathematical modelling were demonstrated. It was established that for mechanisms with closed kinematic chains, the analytical verification of rod stroke and joint angle limits is insufficient. It was empirically confirmed that up to 50% of configurations deemed correct by the mathematical model lead to physical intersections of structural elements in reality. The inclusion of a simulation stage is critically mandatory to prevent mechanism jamming.
- The workspace topology was defined. A 3D gradient reachability analysis revealed a pronounced asymmetry in the workspace, with a «manoeuvrability core» forming in the anterior hemisphere relative to the user. This configuration fully satisfies biomechanical requirements but imposes strict geometric constraints on the design of immersive scenarios: interactive virtual reality objects must be positioned exclusively within the identified zone.
- The computational efficiency of the hybrid architecture was confirmed. Delegating forward and inverse kinematics calculations to a mathematical core and subsequently exporting the data to the multi-threaded C# Job System in Unity enabled the processing of over 750 million static configurations with high efficiency (in under 20 min).

The practical significance of this work lies in the creation of a verified discrete matrix of safe states, which serves as the algorithmic foundation for the kinematic safety layer of the exoskeleton control system. The discrete safe-state matrix can be directly integrated into the real-time control loop as a safety filter: before executing a commanded configuration, the controller verifies its membership in the pre-verified set. This enables the methodology to transition from an offline design tool to an online safety supervisor.

Future research will proceed along several directions. First, experimental validation of the computed workspace using a 3D motion-capture system with the physical prototype will be performed. Second, dynamic models of the pneumatic actuators will be integrated into the digital twin to account for thermodynamic latencies, friction, and valve nonlinearities. Third, algorithms for personalising kinematic limits to individual patient anthropometry and pathology will be developed. Fourth, the approach will be extended to portable exoskeleton configurations. Finally, reinforcement learning techniques will be explored to compensate for residual pneumatic nonlinearities, with the discrete matrix of safe states serving as a formal safety filter that constrains the RL agent’s action space and guarantees collision-free exploration and execution [35].

## Figures and Tables

**Table 1:** Comparison of simulation environments for static collision queries.

Architectural Criterion	Gazebo (DART)	Webots (ODE)	CoppeliaSim (Bullet/Vortex)	MATLAB (Simscape)	NVIDIA Isaac Sim	Unity (PhysX)
Collision Query Mechanism	Strictly coupled with the simulation step. Direct queries require C++ plugin implementation.	Tied to the controller step function. Computes full dynamics.	Built-in API available for instantaneous direct queries.	Absent. Relies on reaction force calculation (penalty functions) during integration.	Tensor-based GPU computations (CUDA/OptiX). Requires Python 3.12 API integration.	Built-in API available for instantaneous direct queries.
Static Check Independence (Time-agnostic)	No (without deep kernel modification). Updates velocities, accelerations, and forces.	No. Simulation time must advance to retrieve sensor data.	Yes. Checks can be performed within a single blocking initialization script cycle.	No. DAE solver requires continuous integration (e.g., ode45/ode15 s).	Possible (if kinematics calculation is isolated), but complex to configure.	Yes. Supports manual forced updates of spatial trees.
Parallel Processing (Multi-threaded Batching)	Difficult. Requires parallel execution of isolated simulation server processes.	No. One controller manages a single physics process.	No. API operates primarily within a single computational thread.	Supports parfor for mathematics, but lacks native detection of complex 3D collisions.	Native. Supports clustering and tensor batching directly on the GPU.	Native. Implemented via the C# Job System.
Potential Performance (Queries Per Second, QPS)	Dependent on simulation step and LCP solver algorithms.	Dependent on simulation step and ODE mass matrix rebuilding complexity.	Tens of thousands per second (dependent on octree or BVH complexity).	N/A (not applied for static polygonal mesh checks).	Millions per second (due to parallel tensor processing on RTX cores).	Hundreds of thousands per second (due to BVH optimisation).
Closed Kinematic Chain Solving (Pneumatic Cylinders)	Supported by physics engine base tools (dynamically).	Supported (dynamically).	Built-in IK solver for complex kinematic chains.	High-performance built-in analytical solvers and optimisers.	GPU-based IK solvers (cuRobo), adaptable for parallel checks.	Requires manual implementation of mathematical apparatus (constraint equations) in C#.

**Table 2:** Scanning parameters.

Parameter	Value
Sphere radius	1.00 m
Spatial grid step	0.01 m
Angle range φ	−45° to +135°
Angle step φ	1°
Total configurations	757,999,583

**Table 3:** Kinematic model constraints.

Parameter	Physical Meaning	Lower Limit	Upper Limit
$x_{p1}$	Maximum pneumatic cylinder rod extension	352.0 mm	502.0 mm
$x_{p2}$		502.0 mm	802.0 mm
$x_{p3}$		277.0 mm	352.0 mm
$x_{p4}$		438.0 mm	638.0 mm
$\theta_{1}$	Shoulder joint rotation (horizontal)	−0.7505 rad (−43°)	+0.7505 rad (+43°)
$\theta_{3}$	Shoulder elevation/depression	−0.6109 rad (−35°)	+0.9250 rad (+53°)
$\theta_{5b}$	Forearm rotation	−0.3491 rad (−20°)	+0.5236 rad (+30°)

**Table 4:** Verification results.

Stage	Number of Points	Percentage of Total	Processing Time (s)
Stage 1. Initial configuration generation	757,999,583	100.00%	702.69
Invalid positions: piston stroke limits	612,203,469	80.77%	
Invalid positions: angular limits	142,739,448	18.83%	
Stage 2. Mathematical model verification passed	3,056,666	0.40%	33.40
Stage 3. Physical collision verification passed	1,627,149	0.21%	376.49

**Table 5:** Dependence of the number of valid configurations on the angle φ.

Angle φ	Math. Verification Passed	Collision Check Passed	Percentage
−35°	26,393	26,387	100.0%
−25°	28,027	21,813	77.8%
0°	30,756	15,289	49.7%
+25°	27,090	11,075	40.9%
+50°	20,135	6922	34.4%
+75°	8217	1717	20.9%
+84°	4313	0	0.0%
+95°	30	0	0.0%

## Data Availability

The raw data supporting the conclusions of this article will be made available by the authors on request.
